# Small Intestinal Ulceration in Two Recurrence-Free Young Patients at 10 Years Postoperatively

**DOI:** 10.7759/cureus.87856

**Published:** 2025-07-13

**Authors:** Taiki Masuda, Yasuko Aoyagi, Sodai Arai, Yu Nishiyama, Mikito Inokuchi

**Affiliations:** 1 Department of Surgery, Musashino Red Cross Hospital, Tokyo, JPN; 2 Department of Surgery, Tokyo Metropolitan Otsuka Hospital, Tokyo, JPN

**Keywords:** abdominal computed tomography, fibrosis, intestinal obstruction, small intestine, stenosis, ulcers

## Abstract

Small intestinal ulcers are occasionally observed in daily medical practice, and although diagnostic abilities have improved in recent years and the disease pathogenesis has been elucidated, recurrence remains common, requiring proper therapeutic intervention. Herein, we report two cases of small intestine ulceration in young patients who did not experience recurrence for a long period of time after appropriate treatment at disease onset. Case 1 was of a 34-year-old woman who had been taking diclofenac sodium. She was diagnosed with intestinal obstruction through abdominal computed tomography (CT). The patient underwent partial resection of the obstructed ileum. Histopathological examination revealed fibrosis in the submucosa, leading to a diagnosis of stenosis secondary to drug-induced small intestinal ulceration caused by nonsteroidal anti-inflammatory drugs (NSAIDs). Postoperatively, the NSAIDs were changed to a selective COX-2 inhibitor (celecoxib). Case 2 was of a 33-year-old man who underwent emergency surgery after an abdominal CT revealed free air. A perforation was found in the small intestine, and the area was resected. Histopathological examination revealed only nonspecific inflammatory findings, leading to a diagnosis of perforation due to a simple small intestinal ulcer. No recurrence was observed in 10 years in both cases. Thus, appropriate management of simple, nonmalignant small intestinal ulcers at initial presentation could be the only treatment needed, with long postoperative recurrence-free periods. These cases demonstrated that proper management of simple, nonmalignant small intestinal ulcers at the initial presentation can be the only treatment needed for long postoperative recurrence-free periods. With an aging society and improved diagnostic capabilities, small intestinal ulcers may become more common in the future. Therefore, the possibility of small intestinal ulceration should be considered when diagnosing ulcerative lesions of the gastrointestinal tract, such as acute abdomen.

## Introduction

Small intestinal ulcers are occasionally encountered in daily medical practice. Although diagnostic capabilities have improved in recent years and the pathogenesis of this disease has been elucidated, recurrence remains common, requiring appropriate therapeutic intervention [1-5]. In this report, we describe two cases of small intestinal ulceration in young patients in their 30s who underwent emergency surgery for acute abdomen and have remained recurrence-free for 10 years after surgery. Written informed consent has been obtained from the patients to publish this paper.

## Case presentation

**Figure 1:**
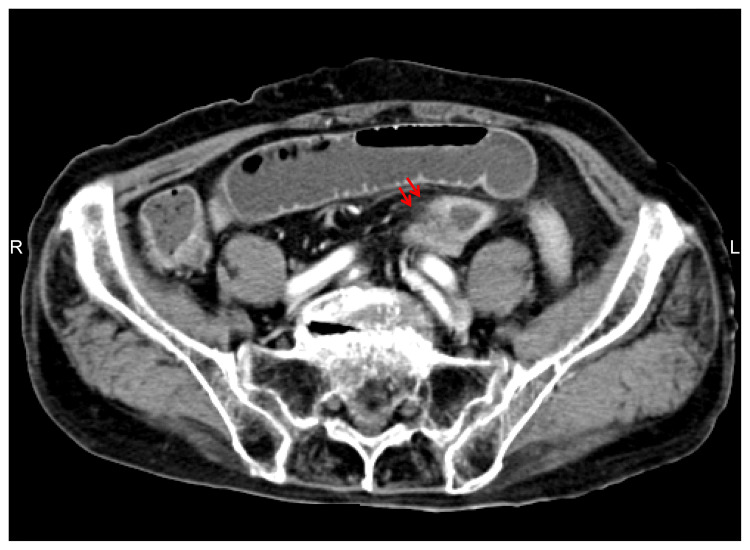
Caliber change (arrow) was revealed on enhanced abdominal computed tomography

Case 1 was a 34-year-old woman whose chief complaints were lower abdominal pain and distension. This patient had been taking diclofenac sodium (50 mg) daily for the past year to treat calcific tendinitis of the left shoulder. The patient had been experiencing intermittent abdominal pain for the past month but had been managing it on her own. One week prior to admission, the patient’s lower abdominal pain and bloating worsened, prompting her to visit our emergency department. At admission, the patient’s vital signs were as follows: body temperature 36.0°C, blood pressure 110/70 mmHg, and pulse rate 96 beats per minute. No anemia or jaundice was observed. Distension, tenderness, and rebound tenderness were noted in the lower abdomen. Blood tests showed a white blood cell count (WBC) of 7,800/μL, C-reactive protein (CRP) of 9.61 mg/dL, and an increased inflammatory response. Fecal tests for pathogenic bacteria were negative. Abdominal and pelvic computed tomography (CT) revealed thickening of the ileal wall and a difference in intestinal diameter at the same site (Figure 1).

Based on the above, a diagnosis of small intestinal ileus was made, and emergency surgery was performed on the same day due to symptoms of peritoneal irritation.

**Figure 2:**
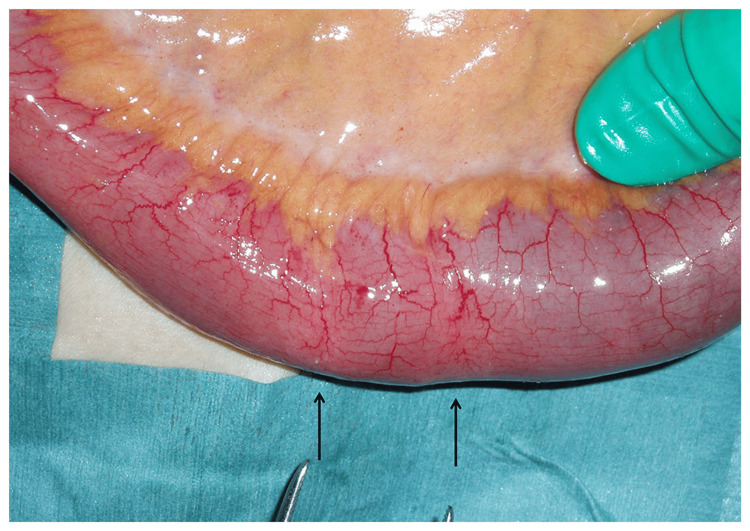
Intraoperative findings: Indurations was detected (arrows) in the ileum 40 cm proximal from the Bauhin valve.

Surgical findings revealed stenosis in the ileum 40 cm upstream from the Bauhin valve, with a ring of induration observed (Figure 2). This stenosis was caused by a small ring-shaped intestinal ulcer. The affected area was partially resected and anastomosed in a single stage.

**Figure 3:**
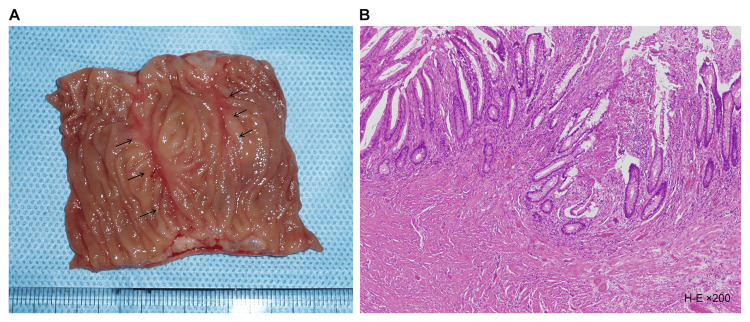
Resected specimen (A) had strictures caused by linear ulcer (arrows), and histological findings (B) revealed submucosal fibrosis.

Macroscopic examination of the resected specimen revealed a ring-shaped ulcer along the short axis, which narrowed toward the center (Figure 3A). Histopathological examination revealed submucosal fibrosis and hyperplastic changes in two mucosal areas. No inflammatory cell infiltration or tumor cells were observed (Figure 3B).

The postoperative course was uneventful, and the patient resumed eating on postoperative day (POD) 3 and was discharged on POD 7. Postoperatively, her nonsteroidal anti-inflammatory drugs (NSAIDs) were changed to a selective COX-2 inhibitor (celecoxib), and a prostaglandin preparation (misoprostol) and a gastric mucosa protective agent (rebamipide) were administered in combination. Furthermore, small bowel endoscopy was performed one year postoperatively, but no abnormal findings were observed. At present, 10 years postoperatively, no recurrence has been observed.

**Figure 4:**
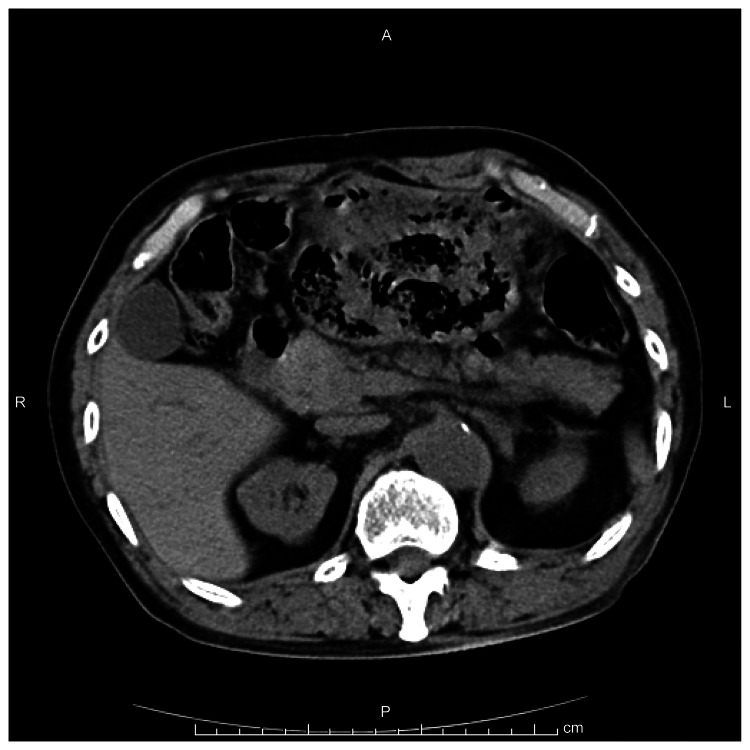
Abdominal computed tomography shows abdominal free air (arrow).

Case 2 was a 33-year-old male patient whose chief complaint was upper abdominal pain. The patient underwent pyloric gastrectomy (Billroth I reconstruction) due to gastric ulcer at the age of 28 years. He suddenly felt upper abdominal pain after dinner and visited our hospital. Vital signs upon admission were a temperature of 38.0°C, blood pressure of 122/76 mmHg, and pulse rate of 90 beats per minute. No anemia or jaundice was observed. The abdomen was board-like and hard, accompanied by tenderness and rebound tenderness. Blood tests revealed a WBC of 14,800/μL, CRP of 3.19 mg/dL (suggesting increased inflammatory response), blood urea nitrogen of 50.2 mg/dL, and creatinine of 2.70 mg/dL (suggesting renal dysfunction). Polymerase chain reaction (PCR) testing for Mycobacterium tuberculosis in gastric fluid was negative. Abdominal and pelvic CT revealed free air in the upper abdomen (Figure 4).

The above results led us to reach a diagnosis of generalized peritonitis due to gastrointestinal perforation, and emergency surgery was performed.

**Figure 5:**
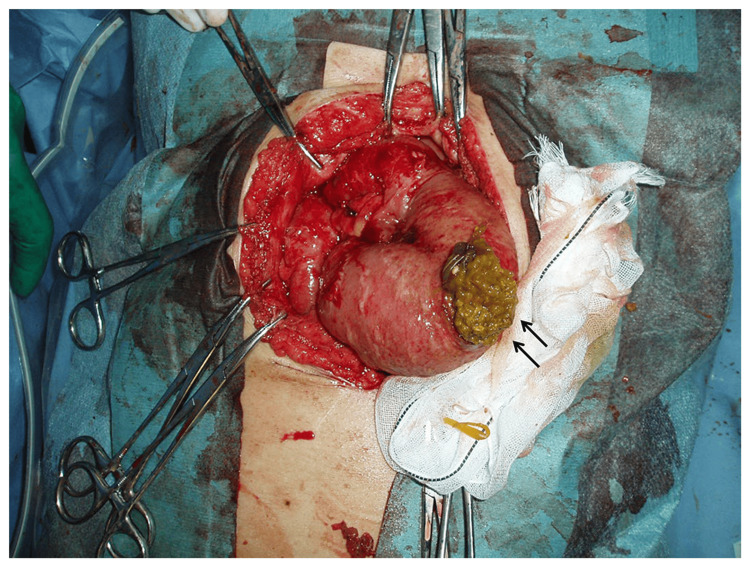
Intraoperative findings: perforation (arrow) in the jejunum 65 cm distal from Treitz ligament was found.

Surgical findings revealed a small intestine perforation of 65 cm from the Treitz ligament toward the anorectal region (Figure 5). No other notable findings were observed in the gastrointestinal tract. A partial resection of the small intestine including the perforation site was performed, followed by a single-stage anastomosis.

**Figure 6:**
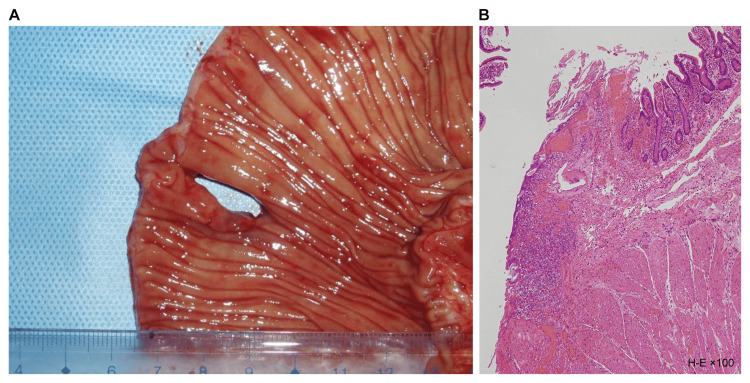
Resected specimen (A) had perforation in the opposite side of a mesentery, and histological findings (B) revealed only the nonspecific inflammation of small intestine.

Macroscopic examination of the resected specimen revealed a punch-out-like perforation on the opposite side of the mesentery (Figure 6A). Histopathological examination revealed only nonspecific acute inflammatory findings, with no necrotic or nonnecrotic granulomas, vascular lesions, parasites, or foreign bodies (Figure 6B).

The postoperative course was uneventful, and the patient resumed food intake on POD 4 and was discharged on POD 8. The patient has been followed up without treatment for 10 years postoperatively, and no recurrence has been observed.

## Discussion

Although small intestinal ulceration is a relatively rare disease, autopsy studies have shown that 8.4% of patients taking NSAIDs had small intestinal ulcers, which was a significantly higher proportion than 0.6% of patients not taking NSAIDs [1]. NSAIDs cause ulcerative lesions in the small and large intestines as well as the upper gastrointestinal tract [2,3]. In recent years, there has been a trend toward the use of sustained-release NSAIDs containing intestinal solvents to mitigate upper gastrointestinal mucosal damage, and it has been suggested that the increased local concentration in the small and large intestinal mucosa induces mucosal damage in the lower gastrointestinal tract [4-6]. Although there are no clear diagnostic criteria for NSAID-induced small intestinal ulcers, the following criteria are generally used to diagnose them [4]: a clear history of prior NSAID use, no prior antimicrobial use, histopathologically negative for pathogenic bacteria, no evidence of specific inflammation, vasculitis, or inflammatory bowel disease, and improvement with NSAID discontinuation and administration of prostaglandins (misoprostol) and gastric mucosal protective agents (rebamipide).

In Case 1, the patient had a one-year history of NSAID use, and a fecal examination for pathogenic bacteria showed a negative result. The ulcer presented as centripetal stenosis, and histopathological examination showed no specific findings. Postoperatively, the patient was treated with a selective COX-2 inhibitor (celecoxib) in combination with a prostaglandin (misoprostol) and a gastric mucous membrane protective agent (rebamipide). Resultantly, the abdominal pain that had been present until then disappeared. These findings led us to reach a diagnosis of NSAID-induced small intestinal ulceration [7]. Previously, the most common symptoms of the disease were diarrhea and bloody stools, and the age of onset was assumed to be bimodal, affecting young and old; however, in recent years, the symptoms have varied and can be found in all age groups [2-8]. This is probably attributed to the fact that younger patients are more likely to suffer from headaches and upper respiratory tract inflammation, whereas the elderly are prone to receive NSAIDs for chronic rheumatoid arthritis and osteoarthritis of the knees. In the lower gastrointestinal tract, the most common sites of occurrence are from the terminal ileum to the ascending colon, although the left side of the colon and the rectum have also been reported [4,9]. It can occur with any type of NSAID and is reported to have occurred with both short-term and long-term administration [7,9]. It is especially important to explain the possibility of intestinal ulcer complications to patients who have been receiving NSAIDs for a long time and to instruct them to visit a medical institution if they have repeated abdominal pain, even if it is mild. Even if no abnormality is detected by upper or lower gastrointestinal endoscopy, the possibility of small intestinal ulceration should be kept in mind, and a reduction or discontinuation of the drug should be considered. In Case 1, the NSAIDs were changed to a selective COX-2 inhibitor (celecoxib) after the surgery, and the patient has not experienced any recurrence for 10 years since the surgery.

In contrast, deep ulcers of unknown cause that histopathologically show nonspecific inflammation are treated as simple small intestinal ulcers, and the number of reported cases has been increasing in recent years [6,10]. A simple small intestinal ulcer is narrowly defined as a chronic corpus callosum ulcer like a peptic ulcer in the ileocecal region, and its lesion image closely resembles that of intestinal Behçet’s disease. Histologically, it is difficult to distinguish between the two, and the concept was proposed to distinguish it from other nonspecific small and large intestinal ulcers [10]. A simple small intestinal ulcer of unknown cause requires its disease concept to be further established. In Case 2, the patient had no history of medications of note and no specific findings on fecal examination or histopathological findings, nor were there any diverticula, ectopic tissue, or foreign bodies. The patient exhibited no symptoms suggestive of Behçet’s disease, and Mycobacterium tuberculosis PCR of gastric juice showed a negative result. No signs of chronic anemia or hypoproteinemia were noted, and serum iron was normal. The above findings led us to reach a diagnosis of a simple small intestinal ulcer. Given that simple small intestinal ulcers have a high postoperative recurrence rate, internal medical therapies such as salazosulfapyridine, steroids, and nutritional supplements are the first choice once a diagnosis is reached [11]. However, in Case 2, the patient developed perforation of the gastrointestinal tract complicated by peritonitis, prompting us to perform emergency surgery. The ulcer was located singly at the perforation site, and only that site was resected. Simple small intestinal ulcers are known to recur frequently [2]. Although small intestinal endoscopy could not be performed as the patient did not give consent, fecal occult blood tests and blood sampling tests are now performed periodically on an ambulatory basis to ensure that recurrence has not occurred. Although it has been 10 years since the surgery, fecal occult blood testing has never been positive, and no anemia has been observed. Consequently, the course has been judged as recurrence-free. The clinical and morphologic characteristics of nonspecific small intestinal ulcers are now being further elucidated by examination of the entire small intestine using capsule and balloon endoscopy.

Because small intestinal ulceration is not very common, it is still not well known to the public and is easily overlooked, especially in young patients. Although small intestinal endoscopy has become widespread in recent years, it is not a common procedure because of its high invasiveness and complexity [2,4]. It is important to keep this disease in mind when diagnosing and treating acute abdomen and follow up appropriately according to the condition to prevent recurrence.

This study has a few limitations. One of the patients underwent surgery 10 years ago, and we were unable to perform advanced testing that is currently available. Another limitation is that we were unable to perform small bowel endoscopy in Case 2 as a surveillance procedure owing to the patient’s noncompliance.

Two cases of appropriately diagnosed and early treatment of small intestinal ulcers are reported. These cases show that it is crucial to follow up a benign disease for 10 years, as this 10-year follow-up allowed us to confirm that there was no recurrence, validating the appropriateness of the initial response.

## Conclusions

Herein, we reported the specific treatment cases we performed on two cases of small intestine ulcers and the long-term follow-up results. No recurrence was observed in 10 years in both patients. These cases revealed that appropriate management of simple nonmalignant small intestinal ulcers at the initial presentation could be the only treatment needed, with long postoperative recurrence-free periods.

Considering the improved diagnostic capabilities and an aging society, small intestinal ulcers may become even more common in the future. Thus, the possibility of small intestinal ulceration should be considered when diagnosing ulcerative lesions of the gastrointestinal tract, such as acute abdomen. Once a diagnosis is made, appropriate initial treatment is desirable.
